# Prevention of *Thelazia callipaeda* Reinfection among Humans

**DOI:** 10.3201/eid2904.221610

**Published:** 2023-04

**Authors:** Marija Trenkić, Suzana Tasić-Otašević, Marcos Antonio Bezerra-Santos, Marko Stalević, Aleksandar Petrović, Domenico Otranto

**Affiliations:** Ophthalmology Clinic University Clinical Center, Niš, Serbia (M. Trenkić);; University of Niš, Niš (M. Trenkić, S. Tasić-Otašević, M. Stalević, A. Petrović);; Public Health Institute, Niš (S. Tasić-Otašević);; University of Bari Aldo Moro, Bari, Italy (M.A. Bezerra-Santos, D. Otranto);; Bu-Ali Sina University, Hamedan, Iran (D. Otranto)

**Keywords:** eyeworm, vector-borne infections, zoonoses, *Thelazia callipaeda*, reinfection, nematodes, Serbia, parasites

## Abstract

*Thelazia callipaeda* is a zoonotic vector-borne nematode that infects and causes eye disease among a wide range of domestic and wild mammals, including humans. We describe an unusual case of reinfection by this nematode in Serbia and call for a focus on preventive measures in endemic areas.

The genus *Thelazia* (order Spirurida, family Thelaziidae) comprises several species of nematode that cause ocular infections in different host mammals, including humans (*1*). Over the past 20 years, the *T. callipaeda* eyeworm has gained interest among the scientific community because several human cases have been reported in countries in Asia and Europe, making this parasite an agent of public health concern (*1*). Adult and larval forms of *T. callipaeda* eyeworms infect the ocular apparatus of a wide range of both domestic (e.g., dogs, cats) and wild (e.g., red foxes, wolves, jackals, bears, lagomorphs) animal species, including humans (*1*–*3*). In Europe, 2 species of drosophilid fruit flies were confirmed to act as vectors of *T. callipaeda* eyeworms: *Phortica variegata*, tested under laboratory and natural conditions (*4*), and *P. oldenbergi* only experimentally (*5*). Thelaziosis should be expected among humans in areas where *T. callipaeda* infection is endemic in animal reservoirs. Absence of preventive measures could be a factor influencing human reinfections in endemic areas. We describe a case of *T. callipaeda* reinfection in a human patient and call for focus on prevention in areas where the parasite and its vectors thrive. 

A man, 41 years of age, living in a small village in the southern part of Serbia contacted an ophthalmologist at the University Clinical Center in Nis, Serbia, because of ocular discomfort. The patient reported a history of clinical thelaziosis caused by *T. callipaeda* infection 5 years earlier. Ophthalmic manifestations were conjunctivitis with increased lacrimation, itching, and sensation of a foreign body in his left eye. During ophthalmologic examination, we removed 11 eyeworms (6 female, 5 male) from the eye and subsequently identified them morphologically as *T. callipaeda* according to an identification key (*6*). The nematodes had a filariform body type with a transversally striated cuticle and a buccal capsule of hexagonal shape. Among female worms, the vulva was located anterior to the esophagus-intestinal junction; male worms had a curved caudal end with 2 asymmetric spicules and precloacal and postcloacal papillae (Table; Figure). 

**Table:** Morphometry of 11 specimens (6 female, 5 male) of *Thelazia callipaeda* from a naturally infected human in Serbia

Measurement	Male		Female
Body length, mm	9.58–12.7 (mean 11.05; SD ±1.16)		13.5–16.5 (mean 14.96; SD ±1.23)
Body width, μm	310–390 (mean 343; SD ±31.14)		360–490 (mean 434; SD ±56.83)
Buccal capsule length, μm	26.4		29.6
Buccal capsule width, μm	25.1		26.4
Nerve-ring from anterior extremity, μm		267.5	373.7
Esophagus length, μm	665.4		719.9
Vulva from anterior extremity, μm	Not applicable		663.2
Left spicule length, μm	1,648		NA
Right spicule length, μm	157.2		NA
Tail length, μm	62.8		72.4

**Figure:**
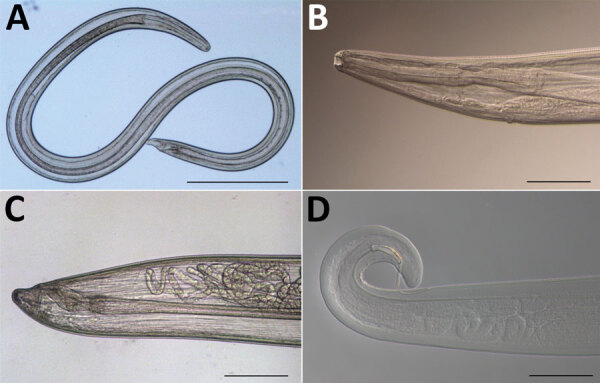
*Thelazia callipaeda* eyeworms collected from the left eye of a man in Serbia. A) Female worm; B) anterior end of adult female; C) posterior end of adult female; D) posterior end of adult male.

To confirm morphologic identification, we extracted genomic DNA from individual worms using a QIAGEN DNeasy Blood & Tissue Kit (https://www.qiagen.com), and performed PCR analysis using the primers NTF (5′-TGATTGGTGGTTTTGGTAA-3′) and NTR (5′-ATAAGTACGAGTATCAATATC-3′), which amplify a 689-bp portion of the mitochondrial cytochrome *c* oxidase subunit 1 (*cox*1) gene. We purified amplified DNA products and sequenced them in both directions using ThermoFisher Big Dye Terminator version 3.1 chemistry in an Applied Biosystems 3130 genetic analyzer with an ABI-PRISM 377 automated sequencer (https://www.thermofisher.com). We analyzed sequences using MEGA version 7 software (https://www.megasoftware.net) and compared them with those available in GenBank using BLAST (https://blast.ncbi.nlm.nih.gov/Blast.cgi). Nucleotide sequences had 100% identity with *T. callipaeda* (GenBank accession no. AM042549.1). In addition, phylogenetic analysis performed by using the maximum-likelihood method based on the Tamura-Nei model showed the representative sequence from our study clustered with other sequences of *T. callipaeda* belonging to haplotype 1 (Appendix), the only haplotype thus far described in Europe. We deposited the nucleotide sequence in GenBank (accession no. OP696980). 

We treated the patient with topical antimicrobials and corticosteroids (rinsing with 3% boric acid 5×/d and topical tobramicin/deksametazon 5×/d). At clinical follow-up 7 and 14 days later, we found no signs or symptoms of eye infection. 

*T. callipaeda* eyeworm prevalence in humans and animals has increased throughout Europe in recent decades (*1*). To date, human thelaziosis has been described in 12 patients from Europe, including a case-patient in Serbia (*7*) reinfected by *T. callipaeda* eyeworms 5 years after an initial case, as in the case we describe here. A related study called for implementing preventive measures, such as vector control and treatment of domestic reservoirs (e.g., dogs), to avoid zoonotic human infection (*8*). In addition, wild carnivore reservoirs, such as red foxes and wolves, should be considered as sources of infection for humans who frequent the same forest areas (*9*). The patient in our study reported that he spent long periods picking mushrooms in the forest, and he exhibited clinical manifestations of thelaziosis during the summer (July), when outdoor activities are most common and the *P. variegata* fruit fly, a *T. callipaeda* eyeworm vector, is most abundant. 

Reinfection in this patient highlights that *T. callipaeda* eyeworms can cause recurrent infection in human hosts, which suggests the potential usefulness of implementing prevention and control strategies in the Balkan Peninsula, where this parasite and its vector and animal reservoirs are spreading (*10*). Moreover, it indicates that inspecting for *T. callipaeda* eyeworms should be part of routine periodic examinations in endemic areas, even among asymptomatic persons. 

Appendix Additional information about a study of *Thelazia callipaeda* infections among humans in Serbia.
