# Supplementary material for: Prevention of Thelazia callipaeda Reinfection among Humans
Source: Emerg Infect Dis. 2023 Apr;29(4):843–5. doi: 10.3201/eid2904.221610 (PMC10045684; doi:10.3201/eid2904.221610)
Supplement: Appendix — Additional information about a study of Thelazia callipaeda infections among humans in Serbia. [file 22-1610-Techapp-s1.pdf]

Article DOI: https://doi.org/10.3201/eid2904.221610

Preventing *Thelazia callipaeda* Reinfection among Humans

EID cannot ensure accessibility for supplementary materials supplied by authors. Readers who have difficulty accessing supplementary content should contact the authors for assistance.

Appendix

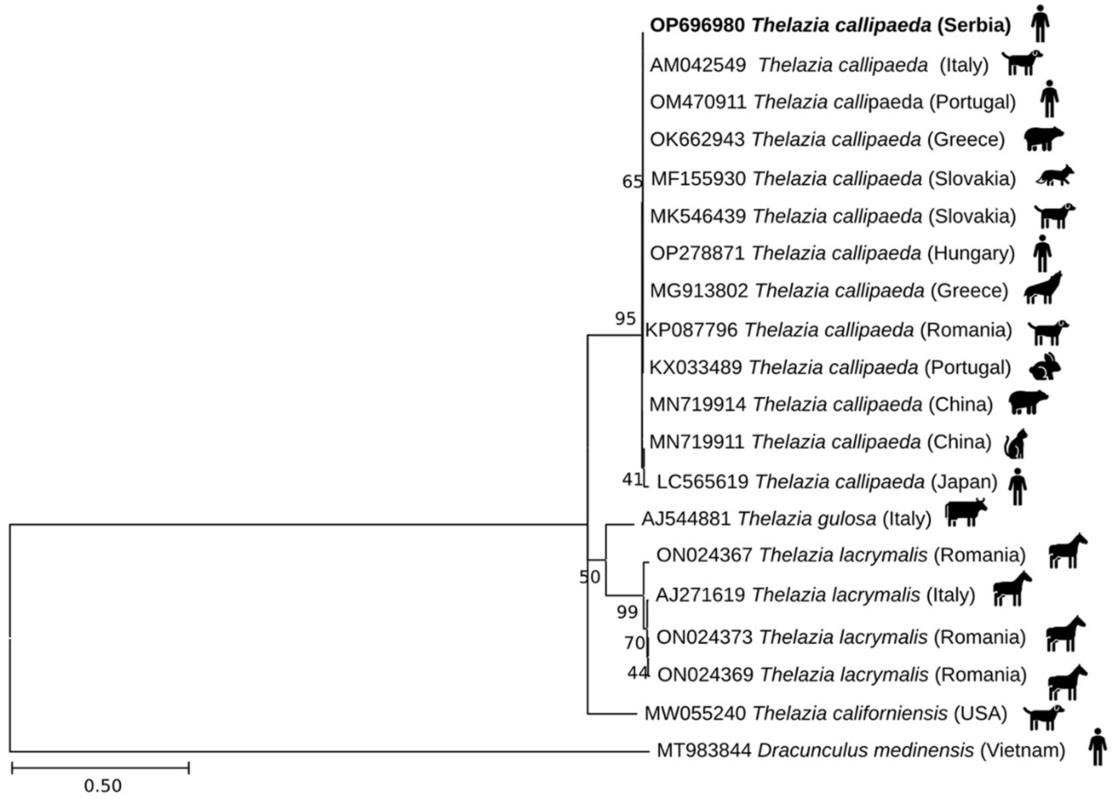

Appendix Figure. Molecular phylogenetic analysis of the *Thelazia callipaeda cox1* gene by maximum likelihood method based on the Tamura-Nei model (*1*) conducted on 2,000 bootstrap replications. The tree with the highest log likelihood (-2128.91) is shown. The percentage of trees in which the associated taxa clustered together is shown next to the branches. Initial trees for the heuristic search were obtained automatically by applying Neighbor-Join and BioNJ algorithms to a matrix of pairwise distances estimated using the maximum composite likelihood (MCL) approach, then selecting the topology with superior log likelihood values. A discrete Gamma distribution was used to model evolutionary rate differences among sites (5 categories [+G, parameter = 0.2595]). The tree is drawn to scale, with branch lengths measured

in the number of substitutions per site. The analysis involved 20 nucleotide sequences. Codon positions included were 1st+2nd+3rd+Noncoding. The representative sequence from this study is in bold. *Dracunculus medinensis* sequence was used as outgroup. Evolutionary analyses were conducted using MEGA7 software.

Reference

1. Tamura K, Nei M. Estimation of the number of nucleotide substitutions in the control region of mitochondrial DNA in humans and chimpanzees. Mol Biol Evol. 1993;10:512–26. PubMed
